# Functional transposition of renal functions to the posterior intestine during maturation in male three-spined stickleback

**DOI:** 10.1038/s41598-025-05513-z

**Published:** 2025-06-20

**Authors:** Yared H Bezabhe, Berkay Paylar, Asmerom Seyoum, Bertil Borg, Per-Erik Olsson

**Affiliations:** 1 The Life Science Center Biology, School of Science and Technology, Örebro University, Örebro, 701 82 Sweden; 2 Department of Zoology, Stockholm University, Stockholm, 106 91 Sweden; 3 Present Address: Department of Environmental Science, Stockholm University, Stockholm, 106 91 Sweden

**Keywords:** Kidney, 11-ketoandrostenedione, Androgen, Solute transporters, Glucose, Molecular biology, Physiology

## Abstract

During the breeding season, the male stickleback proximal tubule of the kidney undergoes hypertrophy. This is due to the synthesis of the nest building protein spiggin, in response to increased levels of 11-ketotestosterone. The increased protein synthesis that is initiated during breeding alters the kidney function and the ability to secrete excess water, to osmoregulate, in fresh water. It has earlier been shown that there exist organ specific differences in transport proteins between mature and non-mature three-spined stickleback. To understand the molecular mechanisms compensating for kidney functions, this study examined transport genes responsible for functional changes between the kidney and intestine. RNA sequencing was performed on castrated and 11-ketoandrostenedione (11KA)-treated male stickleback. Results showed organ-specific responses: 2,549 differentially expressed genes (DEGs) in the kidney and 885 in the posterior intestine, with 210 shared between the organs. Solute transporters, aquaporin 10a and cadherin-17, were upregulated in the posterior intestine but downregulated in the kidney in 11KA treated males. Enrichment analysis revealed distinct biological processes, primarily involving solute transporters, indicating functional adaptation. While amino acid and ion transport were downregulated in the kidney, compensatory transport was observed in the posterior intestine. However, cellular hexose transporters were downregulated in both organs, suggesting a reduction in glucose absorption and passive water diffusion. The present study shows that androgens alter the expression of cellular transporters and redirect functions of the kidney to the posterior intestine. The results also indicate reduced glucose absorption in breeding, male three-spined stickleback.

**Supplementary Information:**

The online version contains supplementary material available at 10.1038/s41598-025-05513-z.

## Introduction

The three-spined stickleback (*Gasterosteus aculeatus*) is a widely studied model organism in evolutionary biology^1,2^. During the reproductive season, male three-spined stickleback undergo physiological changes to support nest building and courtship behavior^3,4^. This includes the development of secondary sexual characteristics such as the display of bright coloration, which is a key factor influencing female mate choice^5,6^. These changes are caused by the gonadal production of 11-ketoandrostenedione (11 KA)^7^which is converted peripherally to 11-ketotestosterone (11 KT), a potent androgen in teleost^8^. 11 KT induces the production of spiggin, an adhesive glycoprotein used for nest building^9,10^. Spiggin is synthesized in the renal secondary proximal tubular epithelial cells and serves as a structural component in nest building. Administration of 11 KA induce renal hypertrophy and spiggin synthesis^10^.

In breeding, male three-spined stickleback, production of spiggin causes hypertrophy of the proximal renal epithelial cells^3,9,11^. The glomerular structure also alters markedly and the glomerular filtration rate decreases, affecting the osmoregulatory function of the kidney^3^. However, breeding males have been shown to begin producing hypotonic fluid using the intestine^6^. This may be achieved by salt secretion accompanied by concurrent transepithelial or paracellular transport of water by aquaporins and/or tight junction claudins proteins^6,12^. Water transport requires that an osmotic gradient has been established. A gradient may be created by the active secretion of fluid containing anions and cations, coupled with a simultaneous reabsorption of Na^+^ and Cl^−13^. Changes in osmotic regulation, genes encoding transport proteins, and ion channels, are of special interest in male three-spined stickleback due to functional changes in the kidney and intestine during breeding.

Considering the possibility of functional switches between the kidney and intestine, it is of interest to identify genes with identical or similar functions in either organ, to understand the potential compensatory mechanisms. This includes the characterization of genes coding for transport proteins in the kidney, and identification of paralogs or isoforms expressed in the intestine that may compensate for the changes in renal function following activation of spiggin production. In a previous study, renal levels of the aquaporin isoforms, aqp1, and aqp8ab, were found to be significantly lower in mature male with hypertrophied kidneys, than in non-mature males^12^. Mature and non-mature males correspond to the 11 KA-treated males and the castrated males in the present study. Higher levels of *Na*^*+*^, *K*^*+*^*-ATPase α−1 subunit* isoforms were observed in mature rather than non-mature male intestines. No differences were observed in aquaporin or tight junction *claudin* proteins^12^. To further determine the regulation of membrane transporters during breeding, a full RNAseq transcriptomics analysis was conducted by comparing castrated male three-spined stickleback with and without 11 KA treatment. Castration makes it possible to study the effect of 11 KA in a controlled manner by removing the source of endogenous androgens and thereby their confounding effects, which could mask subtle changes in the expression of key genes^14^. In the present study, comprehensive gene expression profiles of the kidney, the posterior and the anterior intestine of 11 KA-treated and castrated male three-spined stickleback are presented. While the gills are involved in osmoregulation both in fresh and salt water, it is very unlikely that they could excrete surplus water when the fish are going in freshwater. For this reason, we did not include gills in the present study. Differential regulation of solute and ion transport genes was identified in the kidney and posterior intestine, suggesting a compensatory mechanism in the posterior intestine to support osmotic balance during altered renal function in three-spined stickleback.

## Results

### Effects of surgery and 11 KA implantation on kidney weight

The kidneys in the 11 KA-treated males had hypertrophied and weighed 2.8–4.7% of the body weight, whereas the kidneys in the castrated controls had not and weighed 0.4 to 0.7% of the body weight. Due to high variation of sexual maturity in the sham operated fish, and lack of alignment with either castrated controls or castrated 11 KA treated fish, they were nor included in the study.

### DEG identification

**Fig. 1:**
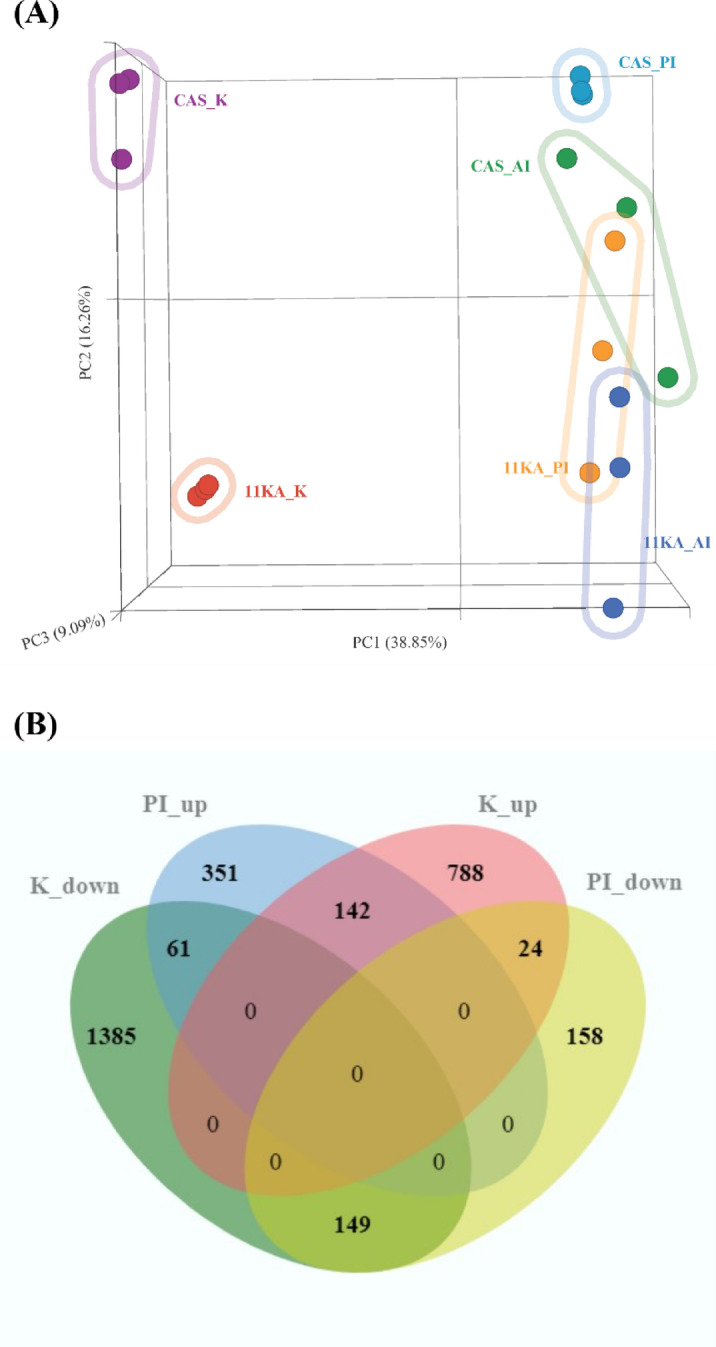
(**A**) Principal component analysis was conducted using normalized transcripts with equal feature contribution. (**B**) Venn diagram shows DEGs in the kidney and posterior intestine of the male three-spined stickleback. Genes showing significant fold changes between 11 KA-treated and castrated males in each organ are shown (*p* < 0.05, FDR ≤ 0.1, log2 FC ≤ −1.2 or ≥ 1.2). CAS_K (Castrated kidney); 11 KA_K (11 KA-treated kidney); CAS_PI (Castrated posterior intestine), 11 KA_PI (11 KA-treated posterior intestine); CAS_AI (Castrated anterior intestine); 11 KA_AI (11 KA-treated anterior intestine); K_up (upregulated in the kidney), K_down (Downregulated in the kidney), PI_up (upregulated in the posterior intestine), PI_down (Downregulated in the posterior intestine).

High-throughput sequencing resulted in 36.4 ± 3.9 million paired-end reads for castrated and 11 KA-treated male three-spined stickleback (*n* = 3). Pre-alignment reads were 151 bp in length with an average read quality of 35.5, a base quality score per position of 36.4, and a Phred score of 33.5 or higher in 97% of reads. Following alignment and normalization, 18,728 genes were identified with a lowest average coverage value greater than one and were consequently used for DEG analysis. PCA analysis showed that 64.2% of the variance between castrated and 11 KA-treated groups could be explained by three principal components (Fig. 1A). 11 KA induced a distinct organ-specific clustering of gene expression profiles, indicating organ-specific responses. Treatment with 11 KA had the highest impact on gene expression in the kidney and the posterior intestine.

Normalized counts were used for differential gene analysis between 11 KA-treated and castrated male samples in each organ using the DESeq2 algorithm. Genes with p-value < 0.05, FDR ≤ 0.1, and log2 fold change of ≤−1.2 and ≥ 1.2, were considered differentially expressed. These thresholds were selected for a broader inclusion of genes to ensure that potentially relevant genes were not missed, which is of particular use when exploring comparative responses across different organs. DEGs among the tissues were compared and presented with a Venn diagram (Fig. 1B). In the two test groups, 2,549 DEGs were identified in the kidney, while 885 DEGs were identified in the posterior intestine. Among these, 376 genes were common to both organs. The number of upregulated genes in the 11 KA-treated groups in the respective organs was 954 and 554. Of these, 142 upregulated and downregulated genes were common to both organs. There were 61 DEGs downregulated in the kidney and upregulated in the posterior intestine. Seventeen of these are solute and ion transporter genes. There were twenty-four genes upregulated in the kidney and downregulated in the posterior intestine but of these, only a monocarboxylic acid transporter, *solute carrier family 16-member 9a* (*slc16a9a*) was a transmembrane transporter. The kidney exhibited significant changes in gene expression, with genes involved in spiggin production showing the highest levels of expression following 11 KA treatment. In contrast, several genes involved in solute and ion balance were downregulated in the kidney (Table 1). In the posterior intestine, expression levels of *aquaporin 10a*, *cadherin-17*, and 22 different ion and solute transport genes, including those downregulated in the kidney, increased in expression. 11 KA induced fewer changes in gene expression in the anterior intestine (Fig S1). Only 11 DEGs were common to the anterior intestine and kidney, and none participated in transmembrane transport (Fig S1A). In comparison, 131 genes were differentially regulated (90 upregulated and forty-one downregulated) by 11 KA treatment in both the anterior and posterior intestine (Fig S1B). This suggests that the anterior intestine is less responsive to 11 KA treatment compared to the posterior intestine and kidney.

**Table 1:** Differentially regulated transmembrane transporters (*p* < 0.05, FDR ≤ 0.1, FC ≤ −1.2 or ≥ 1.2) between the kidney and posterior intestine of 11 KA-treated, breeding, male three-spined stickleback.

Gene symbol	Fold Change (Kidney)	Fold Change (PI)
Kidney downregulated and PI upregulated		
*atp1a1a.2*	−2.39	1.58
*atp1b1b*	−2.86	1.39
*atp2a3*	−1.56	1.87
*slco2b1*	−1.92	1.51
*slc3a2a*	−1.39	1.31
*slc5a1*	−1.54	1.95
*slc7a8a*	−2.39	1.42
*slc9a3r1*	−1.89	1.30
*slc22a7b.3*	−2.88	5.01
*slc26a6 l2*	−1.73	3.95
*slc28a1*	−1.79	1.58
*slc34a2b*	−2.25	2.02
*mpc2*	−1.52	2.12
*mttp*	−5.61	1.62
*vdac2*	−1.40	2.03
Kidney upregulated and PI downregulated		
*slc16a9a*	2.00	−2.32

### qPCR validation

**Fig. 2:**
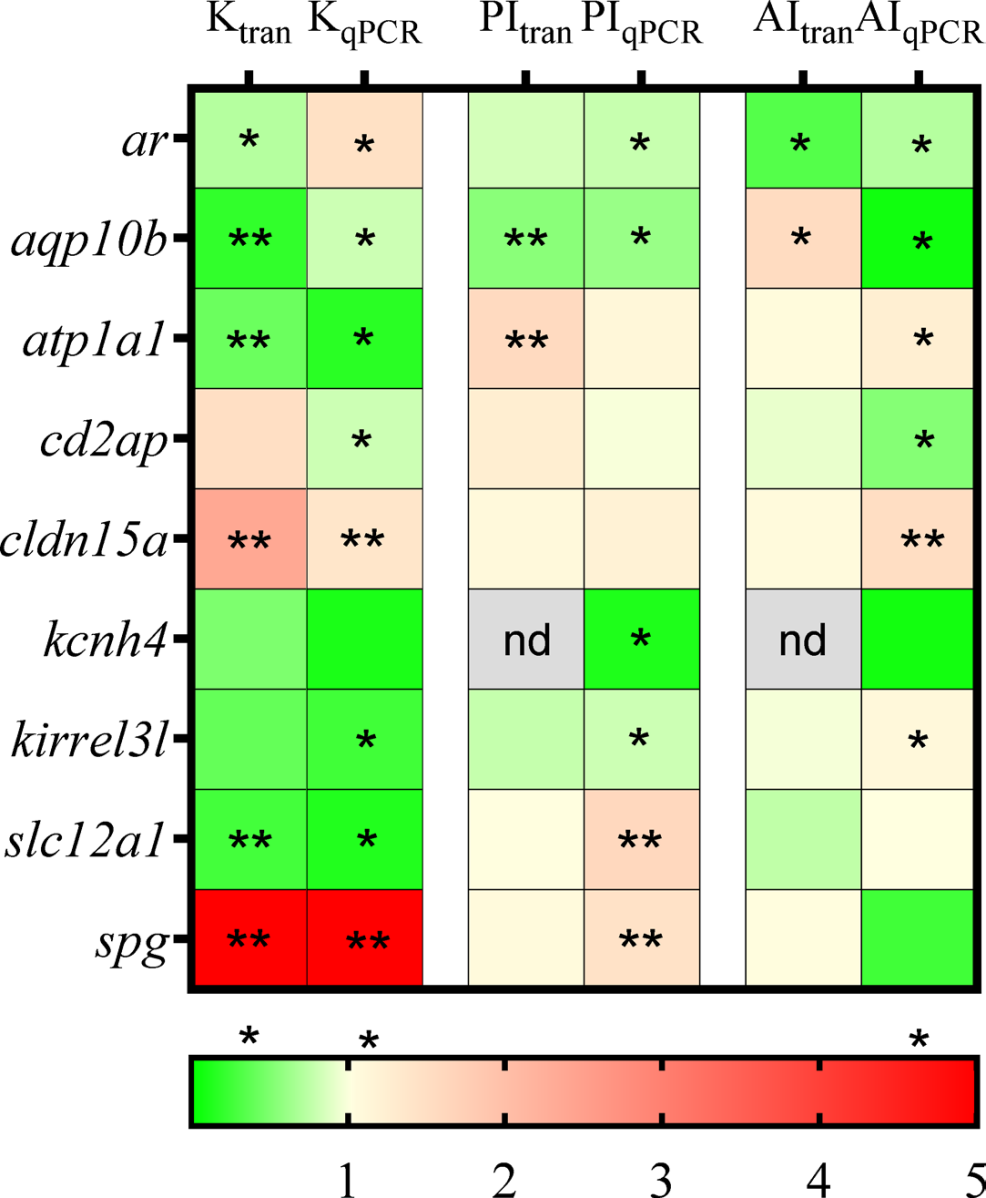
qPCR was conducted to compare the expression levels of the observed DEGs. Genes showing significant fold changes between 11 KA-treated and castrated males in each organ are indicated by red (upregulated) and green (downregulated) (*= *p* < 0.05 indicate difference between castrated and 11 KA implanted fish), nd; not detected, *n* = 4.

qPCR was used to validate the transcriptomics data (Fig. 2). In the validation analysis, 8 out of 10 genes showed similar renal expression pattern in both assays. Only the *ar* and *cd2ap* showed different expression patterns in kidney. In the posterior intestine only *cd2ap* gave different results, with no change in qPCR and a low elevation in transcriptomics. In the anterior intestine *aqp10b* and *slc12a1* gave different results between the two assays. The relative expression levels of DEGs observed in the transcriptomics analysis were confirmed by qPCR, showing a strong correlation between the two analyses. While most qPCR results aligned with RNAseq data, discrepancies for select genes may reflect methodological contrasts. RNA‑seq quantifies reads across all splice isoforms and applies global, library‑wide normalization (e.g., TPM), which can compress extreme expression values, whereas qPCR expression is normalized to reference genes and targets a single amplicon, yielding greater sensitivity at expression extremes. Minor differences in primer binding efficiency and residual biological variability can also affect quantitative estimates. These differences highlight the strength of integrating multiple quantification methods to ensure robust interpretation of transcriptomic results.

### Pathway enrichment analysis of DEGs

**Fig. 3:**
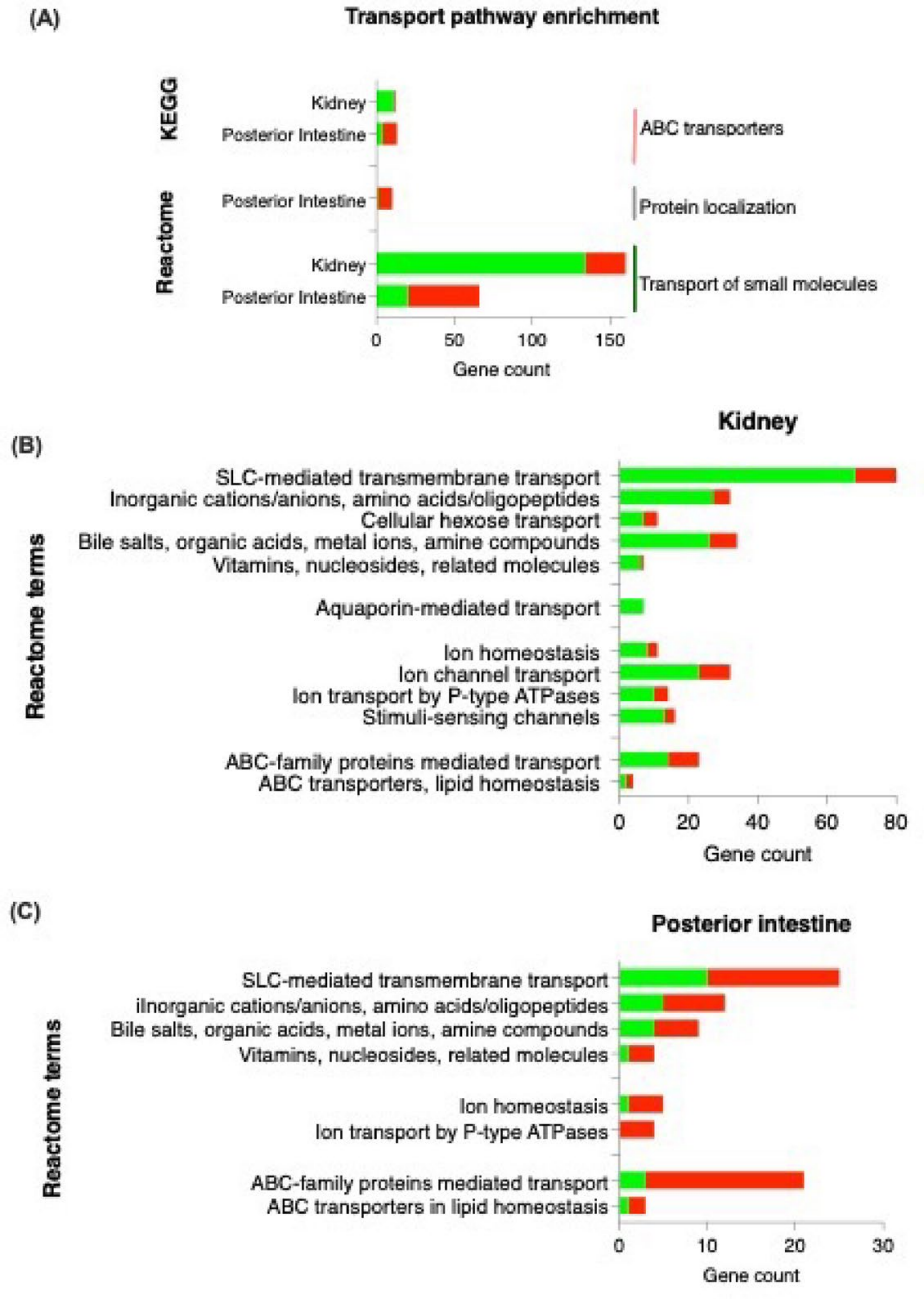
KEGG and Reactome analyses were performed to identify affected transport pathways in the kidney and posterior intestine. The number of upregulated and downregulated DEGs in each tissue are represented by red and green, respectively. Genes showing significant fold changes between 11 KA-treated and castrated males in each organ are indicated by red (upregulated) and green (downregulated) gradients (*= *p* < 0.05, FDR ≤ 0.1, FC ≤ −1.2 or ≥ 1.2).

To identify transport functions affected in 11 KA-treated males, a pathway enrichment analysis was performed with gene enrichment analysis in STRING. Accordingly, ABC transporters were identified in the Kyoto Encyclopedia of Genes and Genomes (KEGG) database, while protein localization, and transport of small molecules were identified to be enriched in the Reactome databases (Fig. 3A). In these pathways, two-thirds of the genes in each pathway were downregulated in the kidney and upregulated in the posterior intestine. In the kidney, 134 (71%) DEGs related to small molecules transport were downregulated, including SLC-mediated transmembrane transport (50%), ion channel transport (17%), and ABC-family proteins mediated transport (10%) (Fig. 3B). SLC transporters are responsible for the movement of solutes across membranes, while ABC transporters play a role in energy-dependent transport mechanisms. In the posterior intestine, upregulation occurred in 46 (30%) DEGs related to small molecules transport, and 9 (90%) DEGs related to protein localization (Fig. 3C). Within the small molecules transport, SLC-mediated transmembrane transport and ABC-family proteins mediated transport were upregulated.

### Gene set enrichment

**Fig. 4:**
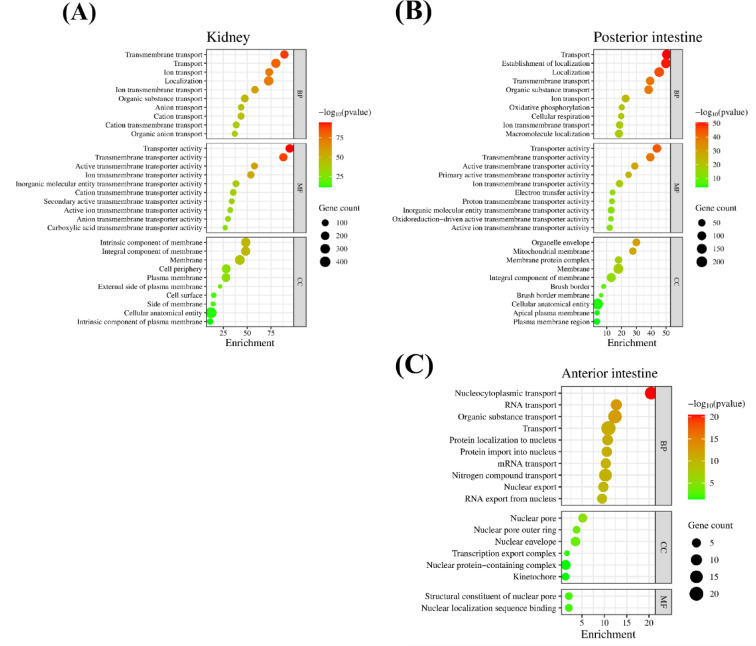
Biological gene ontology was constructed in the STRING database using DEGs for each tissue. (**A**) Kidney, (**B**) posterior intestine, (**C**) anterior intestine. False discovery rate (FDR)-based significance level was used to rank enriched terms. The top ten enriched terms in biological process (BP), molecular function (MF), and cellular component (CC) ontologies are shown. Enrichment is -log10 (FDR). The color and size of each dot represents the significance level of each term and the number of genes that enriched them. Genes showing significant fold changes between 11 KA-treated and castrated males in each organ are indicated by red (highly significant) and green (low significant) gradients.

**Fig. 5:**
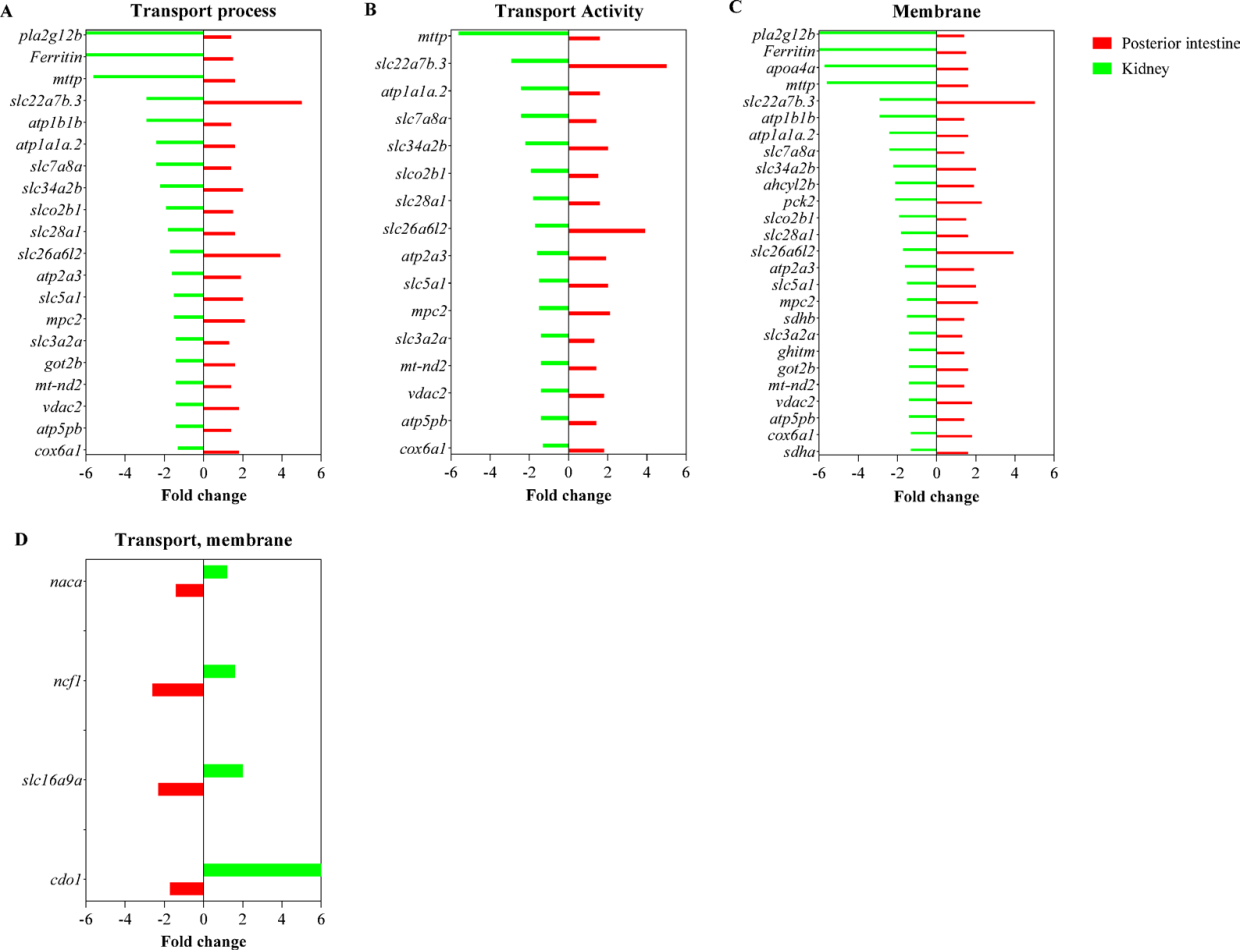
The expression levels of DEGs in enriched transport ontology terms common in the kidney and posterior intestine are shown. (**A**) Transport biological process, (**B**) transport molecular function activity, and (**C**) membrane cellular component terms were highly enriched in tissues with genes that showed distinct differences in expression levels between the kidney and posterior intestine. (**D**) Genes upregulated in the kidney or downregulated in the posterior intestine are shown. Genes showing significant fold changes between 11 KA-treated and castrated males in each organ are shown.

The biological functions, processes, and pathways affected by transmembrane transporters in the kidney and intestine were identified with gene enrichment analysis (Fig. 4). In the kidney, 131 biological processes (BP), 101 molecular functions (MF), and forty-four cell components (CC) transport terms were enriched by 11 KA treatment (Fig. 4A). In the posterior intestine, 96 BP, 69 MF, and 55 CC transport terms were enriched (Fig. 4B). No transmembrane transport GO term was enriched in the anterior intestine (Fig. 4C). Transport/transmembrane transport processes, transporter activity, and membrane cellular component terms, were the most enriched terms in the kidney and posterior intestine. Nitrogen compound transport was the only plasma membrane transport term enriched in the anterior intestine. Most enriched transport-associated terms were found in the kidney and posterior intestine. Subsequent analysis was focused on these two organs. Genes associated with highly enriched transport terms showed distinct expressions between the kidney and posterior intestine (Fig. 5).

### Expression of ion and solute transmembrane transport genes in the kidney and posterior intestine

**Fig. 6:**
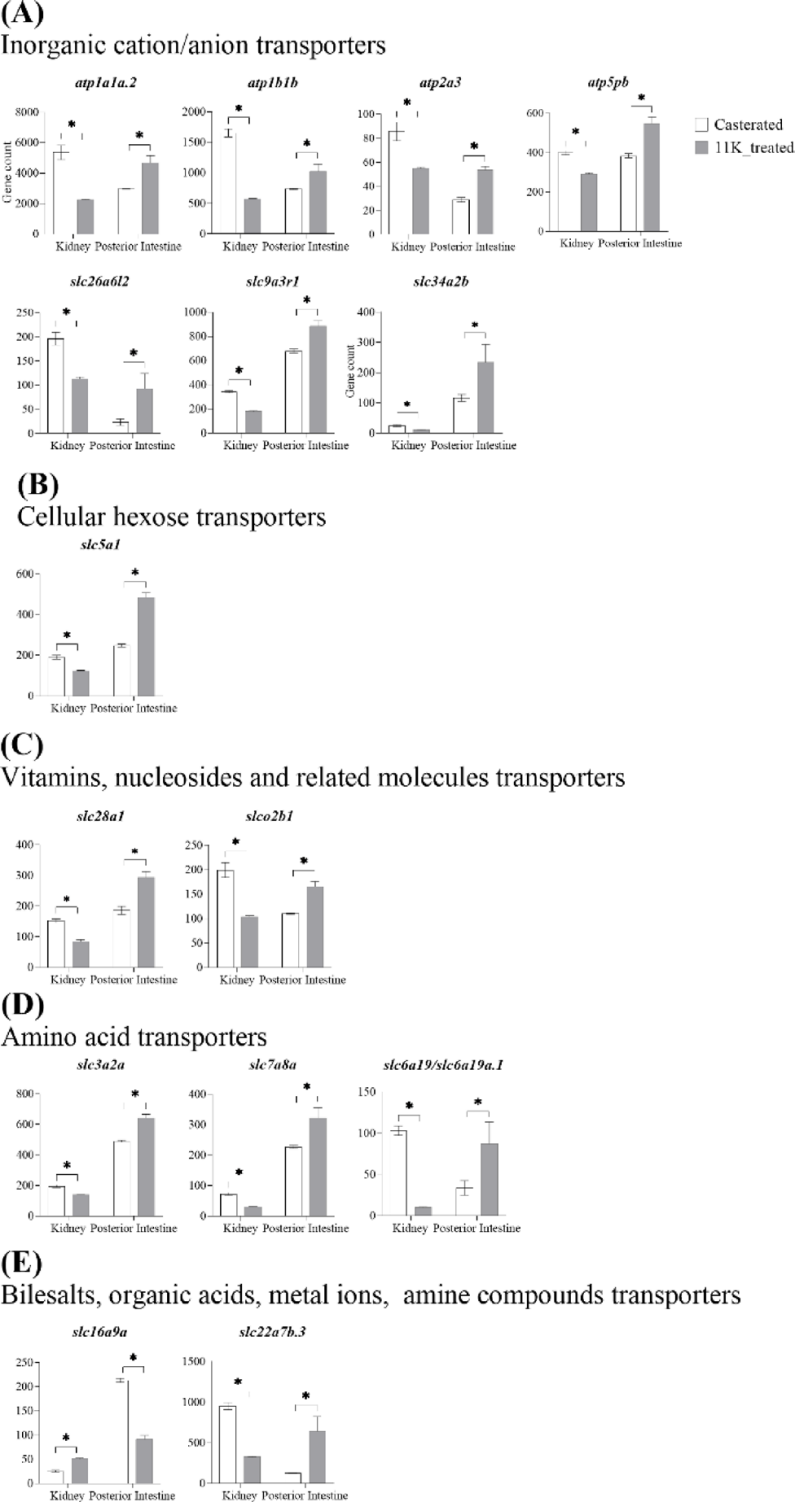
Gene counts of solute transporters in the kidney and posterior intestine. (**A**) Inorganic ion transporters, (**B**) cellular hexose transporters, (**C**) vitamins, nucleosides, and related molecule transporters, (**D**) amino acid transporters, and (**E**) bile salts, organic acids, metal ions, and amine transporters. Genes showing significant fold changes between 11 KA-treated and castrated males in each organ are shown (*= *p* < 0.05, FDR ≤ 0.1, FC ≤ −1.2 or ≥ 1.2).

Fifteen plasma membrane transporter genes were downregulated in the kidney and upregulated in the posterior intestine by 11 KA treatment (Fig. 6; Table 1). These include: seven ions, 1 cellular hexose, 2 vitamins, nucleosides, and related molecules, 3 amino acids, and an organic anion transporter, *slc22a7b.3*, which are within the bile salts, organic acids, metal ions, and amine compounds transporters. A monocarboxylic acid transporter, *slc16a9a*, was downregulated in the posterior intestine and upregulated in the kidney. Among the ion transporters, ATPase Na^+^/K^+^ transporting subunit alpha 1a, tandem duplicate 2 (*atp1a1a.2*) showed the highest expression in the kidney and posterior intestine, more than threefold that of any other transporter (Table S1). ATPase Na^+^/K^+^ transporting subunit beta 1b (*atp1b1b*) had the second highest expression level, indicating the significant role of Na^+^/K^+^ transporting ATPases (NKA) in solute and ion transport in the kidney and posterior intestine. In addition, fourteen genes involved in mitochondrial solute transport, ion transport, and the electron transport chain were also differentially regulated between the kidney and posterior intestine (Fig. 5 and Table S1).

**Fig. 7:**
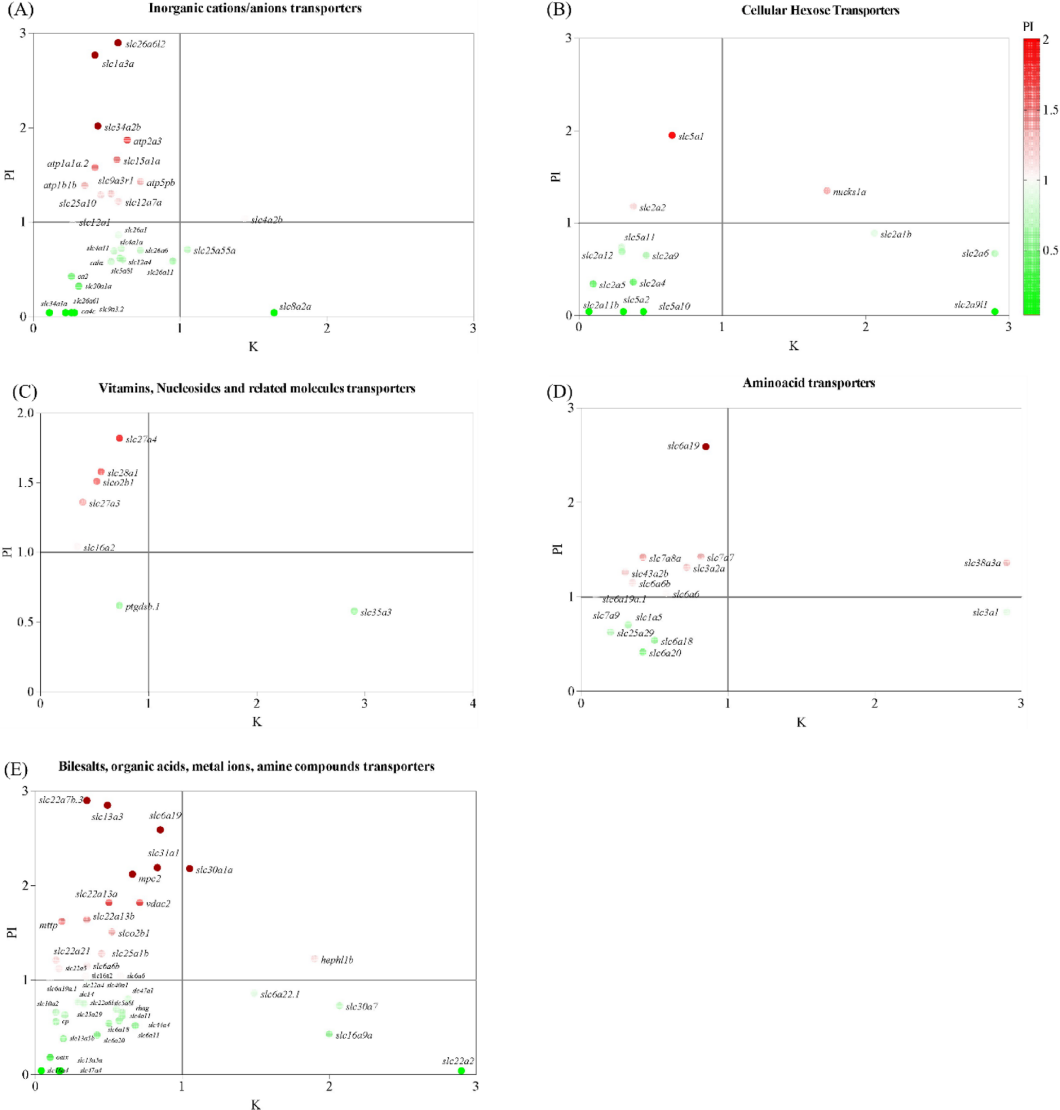
**S**catter plots comparing the regulation of transporters between the kidney and posterior intestine. The ratio of gene counts from the 11 KA-treated group normalized against the castrated group. (**A**) Inorganic cation/anion transporters, (**B**) cellular hexose transporters, (**C**) vitamins, nucleosides, and related transporters, (**D**) amino acid transporters, and (**E**) bile salts, organic acids, metal ions, and amine transporters. Gene regulation in the posterior intestine is shown in the color gradient, with upregulated genes represented by red and downregulated or unexpressed genes represented by green.

Several solute transporters were differentially regulated in the kidney and posterior intestine with exposure to 11 KA, often exhibiting opposite expression patterns between the two organs (Fig. 7 and S2). Many of these genes code for transporters with similar functions (Table S1). A scattering of ion and solute transporter genes were plotted using the count ratio (11 KA-treated versus castrated controls) in the posterior intestine and kidney (Fig. 7). Twenty-four ion and solute transporters that were downregulated in the kidney were significantly upregulated in the posterior intestine (Fig S2). Conversely, fourteen transporters had significantly higher expression in the kidney than in the posterior intestine. The analysis revealed key transporters involved in similar physiological processes but with inverse regulation, highlighting tissue-specific adaptation to 11 KA.

### Expression levels of Aquaporins and tight junction proteins in the kidney and posterior intestine

**Fig. 8:**
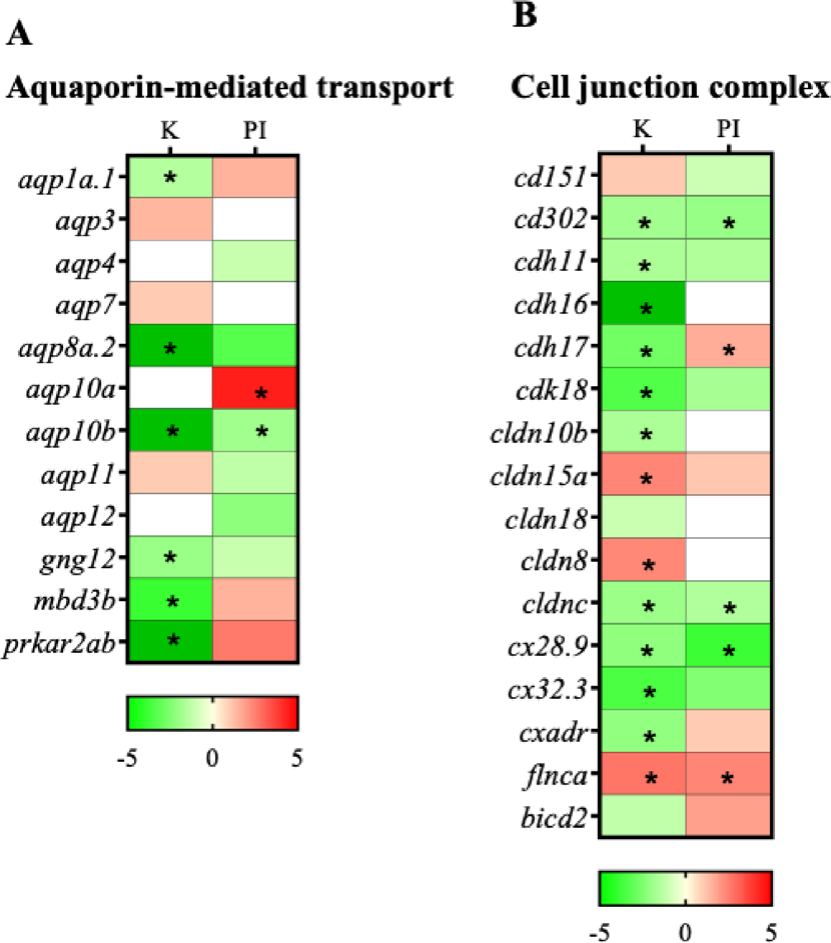
Heatmap showing the differential regulation of analogs and paralogs of (**A**) aquaporins and (**B**) cell junction and vesicle transport proteins. Genes showing significant fold changes between 11 KA-treated and castrated males in each organ are indicated by red (upregulated) and green (downregulated) gradients (*= *p* < 0.05, FDR ≤ 0.1, FC ≤ −1.2 or ≥ 1.2). In each organ, non-expressed genes were shown by white shading. K-Kidney; PI-Posterior intestine.

Eight aquaporin paralogs were expressed in the kidney and posterior intestine but only two paralogs, *aqp10a* and *aqp10b*, showed differential regulation in the 11 KA-treated kidney and posterior intestine (Fig. 8A; Fig S3). A comparison of the expression levels of the different paralogs was made between the kidney and posterior intestine irrespective of 11 KA treatment. Five of these were expressed in the kidney, and the total number of transcripts of *aqp1a.1* and *aqp10b* was high (Table S1). The other paralogs had counts below 30. The *aqp11* transcript level was fifteen times higher in the posterior intestine compared to the kidney. The levels of *aqp10b* and *aqp12* in the posterior intestine were also more than twice the level expressed in the kidney. The levels of *aqp3 and aqp7* were also higher in the kidney than the posterior intestine (data not shown). The paralog *aqp10a* is an important aquaporin downregulated by 11 KA treatment in the kidney and possibly compensated by *aqp10b* in the posterior intestine.

The cell-cell interaction *cadherin-17* (*cdh17*) gene expression was reduced in the 11 KA-treated male kidney and increased in the posterior intestine (Fig. 8B). No differential regulation was observed in claudins between the two organs. Claudins guard tight junctions and determine the permeability of water as well as anion and cation transport between enterocytes. A comparison of expression levels of cadherins and claudin paralogs was made between the kidney and posterior intestine irrespective of 11 KA treatment (Fig S4). Two cadherin paralogs (*cdh11*,* cdh16*) and three proteins involved in tight junction integrity and cell interactions (*cd151*,* cd302*,* cxadr*) were downregulated in 11 KA-treated male kidneys. Among claudins, *cldn8* and *cldn15* had increased expression in 11 KA-treated male kidneys, indicating a function in water secretion in breeding three-spined stickleback. The paralogs *cldn10b*,* cldnc*, *cdk18*, *cx28.9*, and *cx32.3* were downregulated in both the 11 KA-treated male kidneys and posterior intestine.

## Discussion

In an earlier study on breeding three-spined stickleback, reduced levels of solute transporters in the proximal tubules of the kidney were observed to be compensated by the same transporters or transporters with similar functions in the intestine^12^. Using full RNAseq transcriptomics analysis, this compensatory relationship was confirmed. Additional transporters across broader solute types exhibiting opposite regulation patterns were also identified, emphasizing tissue-specific adaptation to 11 KA treatment.

The comparative transcriptome analysis of 11 KA-treated and castrated groups showed that two basolateral Na^+^/K^+^ transporting ATPases (NKA), *atp1a1a.2* and *atp1b1b*, and the multifunctional anion exchanger, *slc26a6 l2*, were downregulated in the kidney and upregulated in the posterior intestine of treated male three-spined stickleback. These genes were also overrepresented in transport processes, activity, and intrinsic/integral parts of plasma membrane gene ontology (GO) terms in the ontology analysis. In contrast, *slc12a1* and *slc12a4*, which participate in apical Na^+^, Cl^−^ reabsorption, were downregulated in the kidney without significant changes in the posterior intestine, indicating a reduction in apical Na+/Cl⁻ reabsorption.

Previous studies have shown that NKAs regulate salt secretion and reabsorption in the kidney and posterior intestine, contributing to the osmolarity of luminal fluid^12,15,16^. Additionally, *slc26a6 l2* in combination with *slc12a1*, *slc12a2*, *slc12a3*, apical cystic fibrosis transmembrane conductance regulator (CFTR), and paracellular Na^+^ leakage, has been implicated in apical Na^+^, Cl^−^ reabsorption and organic anion secretion in marine teleost fish and the anadromous Atlantic salmon^13,17^. The differential regulation of *atp1a1a.2*,* atp1b1b*, and *slc26a6 l2* observed in this study aligns with these findings, further supporting their role in transmembrane salt transport. However, the downregulation of *slc12a1* and *slc12a4* in the kidney differs from previous observations, which may be attributed to differences in methodologies^12^. Unlike the qPCR-based assessments of target-specific genes employed in the previous studies, RNA-seq provides a comprehensive (sum of all transcripts) overview of gene expression, capturing variations across the entire transcriptome. This broader approach may uncover regulatory patterns that were not identified in gene-specific analyses, such as subtle or opposing trends in distinct regions of an organ. Taken together, the upregulation of *atp1a1a.2*,* atp1b1b*, and *slc26a6 l2* in the posterior intestine indicates a compensatory adaptive mechanism in male three-spined stickleback that enhances transmembrane salt transport. This adaptation facilitates the production of hypotonic luminal fluid during breeding, supporting osmoregulation under reproductive conditions.

The Na^+^/H^+^ exchanger *slc9a3* (*nhe3)* is another renal apical membrane acid-base regulator that affects Na + mediated transmembrane solute transport in freshwater rainbow trout and three-spined stickleback^12,18^. In this study, a splicing variant, *slc9a3.2*, was downregulated in the kidney but not expressed in the posterior intestine. However, its regulator, *slc9a3r1* (*nherf1a*), was downregulated in the kidney and upregulated in the posterior intestine. The paralog s*lc9a3r1* is described in teleost as an apical plasma membrane protein involved in both protein localization to a membrane and sodium ion homeostasis in the kidney^19^. The differential regulation of s*lc9a3r1* but not *slc9a3/nhe3* in this study indicates the existence of an unknown Na^+^/H^+^ exchanger. As a result, these findings strongly support previous observations of an established and well-regulated Na^+^, Cl^−^ reabsorption system in the posterior intestine responsible for hypotonicity.

Reabsorption of glucose in the proximal tubules has previously been suggested to be affected by 11 KT treatment^13,20^. In the present study, 10 of the 13 differentially regulated apical and basolateral Na^+^/glucose symporters were downregulated in the kidney. One of these genes, *slc5a1a*, was upregulated in the posterior intestine. Paralogs such as *slc5al* reabsorb glucose along with Na^+^ in the proximal tubule and intestine of several other fish species^13,20,21^. In addition, *slc5a1-slc5a4* and *slc5a9-11* are involved in glucose homeostasis and uptake in the intestine, muscle, liver, and kidney of teleost^22,23^.

The only 11 KA upregulated transporter genes in the kidney were a low-capacity glucose and fructose transporter, *slc2a9 l1* and facilitated glucose transporter, *slc2a1b*. SLC2 A9 (GLUT9) is a high-capacity, urate-reabsorbing intestinal transporter with residual high affinity for glucose and fructose transport^23–26^. The present study is the first to detail the expression levels of sodium-glucose transporters in 11 KA-treated three-spined stickleback. Following 11 KA treatment, the Na^+^-D-glucose co-transporter, *slc5a1*, demonstrated functional transposition as it was downregulated in the kidney and upregulated in the posterior intestine. However, all facilitative glucose transporters (GLUT, SLC2) involved in glucose absorption in the posterior intestine were downregulated, suggesting a possible reduction in glucose absorption^27–29^.

11 KA treatment also affected amino acid and ion transmembrane transporters. In the kidney, thirteen out of seventeen differentially regulated amino acid transport genes were downregulated. Two neutral and basic amino acid transporters, *Slc3a1* and *slc38a3a*, one gamma-aminobutyric acid (GABA) transporter, *slc6a22.1*, and one oligopeptide transporter, *abcb9*, were upregulated. The transmembrane transport of amino acids in the kidney provides the necessary building blocks for spiggin synthesis. Eight amino acid transporters were upregulated in the posterior intestine, including two which were contrarily downregulated in the kidney. The upregulated genes of the posterior intestine have similar functions to those downregulated in the kidney, possibly compensating for decreased reabsorption in the proximal tubules. For this reason, amino acid reabsorption may not be affected by 11 KA treatment in breeding males.

Organic anion and cation transmembrane transport is also affected by 11 KA treatment in breeding male three-spined stickleback. Renal transport of endogenous metabolites and exogenous toxins are crucial processes in the regulation of energy metabolism and acid-base homeostasis^30,31^. In teleost fish, ion exchangers and apical Na^+^, Cl^−^ reabsorption, coupled with the secretion of organic anions and cations, maintain the membrane solute gradient, composition and osmolarity of urine^13,17^. In the present study, twenty-one out of twenty-eight differentially regulated organic cation/anion/zwitterion transporters, including organic anion (14) and cation (7) transporters, were downregulated in the kidney of 11 KA-treated, male three-spined stickleback. Genes down-regulated in kidney and upregulated in posterior intestine included the bile acid transmembrane transporter, *slco2b1*, microsomal triglyceride transfer protein large subunit (*mttp*), mitochondrial pyruvate carrier 2 (*mpc2*), and voltage-gated monoatomic anion channel (*vdac2*). Kidney genes involved in the exchange of organic anions and cations such as urate, orotate, and nicotinate, were not compensated in the present study, but have been shown to affect secretion and possibly osmolarity of luminal fluid^30,32–34^. Decreased expression of organic anion and cation transporters in the kidney, and a lack of compensation in the posterior intestine, suggest a reduction in secretory functions during breeding.

Aquaporin-mediated water transport from cells into the lumen is tightly regulated by tight junction proteins, claudins, and cadherins of fish^13,35–37^. There are indications that intestinal tight junctions are responsible for the possible occurrence of paracellular osmotic water flow in breeding males^3^. Taken together, some solute transporters involved in intestinal absorptions may also affect ionic and solute balances, which could serve as an effective compensatory mechanism for loss of key renal functions in breeding, male three-spined stickleback.

The proximal tubule has been shown to be responsible for NaCl and water transport^13,38^. Apical Na^+^ reabsorption may involve Na^+^/H^+^ exchanger 3 (NHE3) and solute-carrier family 5 (SLC5 A, Na^+^/glucose cotransporter)^13,18^. Cl^−^- reabsorption is mediated by the solute carrier family 26-member 6 (SLC26 A6)^13,17,39^. It has been proposed that NaCl and water transport in the proximal tubules involve uncharacterized basolateral Na^+^-K^+^-ATPase (NKA) isoforms^15,16^. NKAs are P-type ATPases that utilize the energy from ATP hydrolysis for active ion transport of one or two ion species across the plasma membrane^16^. Based on findings from killifish experiments, basolateral secretory-type NKCC1 (SLC12 A2) drives water secretion (through aquaporins and/or tight junctions) in the proximal renal tubules along with NaCl and Mg^2+15,38^. Based on this observation, secretion of hypertonic fluid from intestinal cells may be driven by basolateral Na^+^- K^+^-ATPase ion-transporter (NKA) and secretory-type Na^+^, K^+^, 2 Cl^−^ cotransporter NKCC1, coupled with the apical chloride channel (CFTR)^35^. This creates an osmotic gradient that drives water from cells into the lumen via aquaporins or tight junctions^37^. As a result, hypotonicity can be created in the posterior intestine through the active reabsorption of salt by NKA and apical absorptive-type NKCC2 (SLC12 A1) and Na^+^ (K^+^), Cl^−^ -cotransporters (NCC, SLC12 A3 coupled with basolateral chloride channels^12,15^. However, functional studies are needed to confirm these mechanisms in the posterior intestine of breeding male stickleback.

Only two isoforms of *aqp10* showed differential regulation between the kidney and posterior intestine after 11 KA treatment. While *aqp10a* was downregulated in the kidney, *aqp10b* showed increased expression in the posterior intestine. None of the other six expressed isoforms showed differential regulation between the organs. Two vesicle-mediated transporters, cx28.9 (kidney and posterior intestine) and cx32.3 (kidney), were also downregulated. Three of these paralogs were previously identified as true water aquaporin (*aqp1a/aqp1a.1*) and aquaglyceroporins (*aqp3a*, *aqp10a*, *aqp10b*)^12,40,41^. Previous work has shown that the levels of *aqp1a.1* are lower in the kidneys of mature (breeding) males than in non-mature males, while *aqp1a* is expressed in moderate to low levels in most tissue, while no difference in *aqp10b* have been observed^12^. Even though it is difficult to make direct comparisons due to methodological differences, the lower levels of *aqp1a.1* observed in 11 KA-treated males are consistent with previous findings^12^. The observed difference in *aqp10b* levels could be due to the use of castration in the present study instead of the non-mature males used in the previous study. In non-mature males, low levels of endogenous androgens may mask subtle changes in the expression of key genes^14^.

Enterocyte tight junctions have been implicated in extra-renal water secretion mechanisms hypothesized to be present in breeding males^3^. These junctions play a central role in paracellular osmotic water flow, a process primarily governed by claudins, which regulate permeability to water, anions, and cations between enterocytes^42^. Supporting this hypothesis, there are studies suggesting the involvement of intestinal tight junctions in osmotic water transport^13,37,43,44^. However, neither this study nor earlier work^12^ identified any significant differential expression of claudins between the kidney and posterior intestine in breeding male three-spined stickleback. The lack of variation in claudin expression between these tissues aligns with previous findings and suggests that other proteins may contribute to tissue-specific adaptation during breeding.

Interestingly, among three cadherin isoforms identified in the kidney and posterior intestine, only *cdh17* exhibited significant downregulation in the kidney and significant upregulation in the posterior intestine of 11 KA-treated males. This differential regulation, combined with GO ontology and pathway analysis linking *cdh17* to transport terms, highlights its potential involvement in intestinal water transport. These findings suggest that while claudins may provide a stable structural framework for tight junctions, the dynamic regulation of *cdh17* could represent a complementary mechanism facilitating tissue-specific osmotic adaptations during the reproductive period.

Cadherin-17, also known as liver-intestine cadherin (LI-cadherin), was highly upregulated in the posterior intestine of 11 KA-treated males, compared to castrated males. Cdh-17 belongs to the 7D-cadherin subfamily and is localized to the lateral plasma membrane of enterocytes, distinct from adherent junctions^43,44^. A study in CACO2 cell monolayers demonstrated that this specific positioning enables the protein to narrow the intercellular cleft, facilitating water reabsorption by supporting the buildup of an osmotic gradient^44^. Since cadherin levels were not assessed in previous studies on three-spined stickleback, direct comparisons are not possible. However, the marked upregulation of *cdh17* in breeding males highlights its potential contribution to the production of hypotonic luminal fluid in the posterior intestine.

In summary, the differential regulation of solute and ion transporters, coupled with changes in tight junction-associated genes, suggests a functional transposition of renal transport mechanisms to the posterior intestine in breeding male three-spined stickleback. This adaptation may enable males to maintain solute and water balance during the reproductive season when kidney function is constrained by other physiological demands. Specifically, the upregulation of the basolateral Na+/K + ATPases (*atp1a1a.2* and *atp1b1b*) and the multifunctional anion exchanger, *slc26a6 l2*, in the posterior intestine, support enhanced transmembrane salt transport. In addition, the reduced expression of cellular hexose transporters suggests that absorption of glucose in the intestine leading to increased luminal solute levels may create a gradient resulting in passive water secretion.

The lack of differential expressions of claudins suggests a stable tight junction framework, while the pronounced upregulation of *cdh17* highlights its potential role in mediating compensatory water transport via tight junction remodeling. Together, these findings provide molecular evidence for the secretion of hypotonic luminal fluid by the posterior intestine, thereby complementing renal function and contributing to osmoregulation during breeding. The comprehensive transcriptome approach employed in this study offers valuable insights into organ-specific adaptations and underscores the complex interplay of solute and water transport mechanisms in breeding males.

## Methods

### Animal maintenance and experimental setup

Adult three-spined stickleback males were caught in Öresund, southern Sweden, in winter. In the laboratory, they were kept under a long photoperiod of LD 16:8 and 16 °C and at a salinity of 0.5%. Thirty-two fish were anesthetized using 250 mg/L MS222 buffered with sodium bicarbonate. Operation of the fish was made by small lateral incisions into the abdominal cavity, and testes were removed using forceps. Silastic tubes ID 0.64 and OD 1.19 mm, length 10 mm, containing cacao butter alone or cacao butter with 1% 11-ketoandrostenedione (Sigma), were implanted intra peritoneally and the incisions were closed with sutures. The operated males, 10 castrated controls and 10 castrated 11 KA implanted fish, and 12 sham-operated fish with testes remaining, were put into a 1200-l aquarium 15 to 60 min following surgery with 0.5% salinity water under 16 °C and LD 16:8. The fish were considered fully recovered when they were swimming around normally. One sham operated fish did not survive the experiment. The water was constantly aerated and filtered. The bottom was covered with sand and ceramic tubes served as hiding places. The fish were fed bloodworms or mysids, daily, except on the operation day, the day after operation, and on the dissection day. Following surgery, the temperature was raised over two days to 20 °C using immersion heaters. Eleven days after surgery the fish were moved to a similar aquarium with 0.25% salinity, and subsequently to fresh water (Stockholm tap water with a hardness of 4–6 dH) 6 days later. Under these conditions, most sham-operated and 11 KA-treated fish attained breeding colors, whereas the castrated controls did not. Two weeks after transfer to fresh water the fish were euthanized using 250 mg/L MS222 buffered with sodium bicarbonate followed by decapitation and destruction of the brain. The kidney samples did not include the head kidney, which is separated from the trunk kidney in the three-spined stickleback and do not contain nephrons. The intestine lumen was wiped clean with cotton tops and a narrow border zone between the anterior and posterior intestine was removed. The kidney and the anterior and posterior intestines from three fishes from each treatment (18 samples in total) were divided longitudinally, and half were placed into tubes with 500 µL RNAlater and stored at −80 °C. The experimental protocol was approved by the Stockholm Animal Experiment Ethical Board 4730 − 2019, and performed in accordance with the relevant guidelines and regulations. All methods are reported in accordance with with ARRIVE guidelines^45^.

### RNA isolation, sequencing, and transcriptomic analysis

The samples were homogenized in Tri Reagent (Sigma) and RNA was extracted from the organs using a Direct-zol RNA Kit (Zymo Research, USA) and included DNase treatment of the samples. RNA concentrations were determined spectrophotometrically using a DeNovix DS-11 spectrophotometer (Wilmington DE, USA), and RNA quality was determined by agarose (1%) gel electrophoresis in 1x TBE and by the ratio of absorbance 280/260 nm (1.8–2.0) and 260/230 nm (2.0–2.2). RNA sequencing was done at GATC Biotech/Eurofins. RNA samples with an RNA integrity number greater than or equal to 8 were used for sequencing. Sequence libraries were prepared from 1 µg of total RNA using a NEBNext Ultra RNA Library Prep Kit. The libraries were sequenced in a HiSeq2500 with a read length of 2 × 51 reads.

Sequence analysis was done with Partek Flow (St. Louis, Missouri, USA). The sequence quality of raw reads was assessed with pre-alignment QA/QC. Based on the quality score, sequences were trimmed from the three prime ends and the average Phred score was determined for trimmed reads. Trimmed reads were aligned to the *G. aculeatus* genome from the Ensembl database (BROAD S1, assembly 81) using STAR aligner (Version 2.5.3a) with default settings. Quality of alignment was performed using the post-alignment QA/QC tool (Table 2). Genes were quantified to the annotation model with default settings including a minimum read of 10. Counts were normalized to counts per million (CPM) and then 1.0E-4 was added according to Partek’s recommendation. Normalized counts were split by organ and further analyzed for differential expression between castrated and 11 KA-treated males using DESeq2 algorithm with default settings. Genes with p-value < 0.05, FDR ≤ 0.1, and log2 fold change of ≤−1.2 & ≥1.2 were considered differentially expressed between castrated and 11 KA-treated male groups.

**Table 2:** RNA-sequence post-alignment quality check for the read pairs.

Sample	Total reads	Total alignments	Aligned	Avg. coverage depth	Avg. length	Avg. quality	%GC
M11 KAAI	32,623,859	68,127,129	92.14%	81.15	150.92	35.94	50.73
M11 KAAI	40,143,289	84,327,028	90.79%	101.66	150.92	35.97	51.04
M11 KAAI	35,524,786	75,082,079	93.11%	101.11	150.93	35.93	50.89
M11 KAK	35,364,773	73,281,139	91.74%	90.35	150.93	35.99	52.12
M11 KAK	39,038,920	81,931,418	92.80%	93.42	150.92	35.95	50.95
M11 KAK	33,120,945	69,885,917	93.49%	82.26	150.92	35.93	50.84
M11 KAPI	33,963,544	71,774,462	93.24%	90.31	150.93	35.93	51.30
M11 KAPI	32,201,952	67,445,732	92.60%	78.67	150.92	35.86	50.71
M11 KAPI	31,824,928	65,490,714	91.68%	87.45	150.93	35.96	52.08
MCASAI	35,708,176	75,271,097	91.04%	82.38	150.92	35.88	49.96
MCASAI	49,149,117	105,522,813	92.26%	126.72	150.92	35.86	50.82
MCASAI	37,237,398	79,784,610	92.56%	85.98	150.92	35.91	50.86
MCASK	37,124,416	78,617,029	93.15%	79.37	150.92	35.97	50.70
MCASK	37,226,506	78,551,517	92.42%	79.74	150.92	35.98	50.33
MCASK	36,922,882	78,166,490	93.34%	78.29	150.92	35.95	50.37
MCASPI	35,498,449	76,054,550	92.92%	94.48	150.92	35.92	50.40
MCASPI	35,065,726	74,554,789	91.06%	95.40	150.92	35.93	51.38
MCASPI	38,454,568	76,684,239	87.33%	92.72	150.92	35.91	50.47

### qPCR validation

To validate the transcriptomics analysis, primers were designed for ten genes and analyzed with qPCR using separate samples (Table 3). cDNA was synthesized from RNA (1000 ng) from 3 biological replicates using the qScript cDNA synthesis kit (Quanta Biosciences, USA) according to the manufacturer’s instructions. Expression of genes was performed with qRT-PCR using qPCRBIO SyGreen Mix Lo-ROX (PCR Biosystems, USA) using the CFX384 Real-time PCR detection system (Bio-Rad, USA). Amplification was done with thermal cycling profiles of the initial denaturation step at 95 °C for 2 min followed by 35 cycles of 95 °C for 5 s and 60 °C for 30 s. Expression ratios were calculated based on the ΔΔCt method^46^ using elongation translation factor 1α (eef1α) as for normalization of gene expression. Eef1α was selected as reference gene following determination of stability of expression between samples and treatments.

**Table 3:** Primers used for validation.

Gene	Sequence	Ensembl gene ID
*eef1α*	F: CATTGTCACTTACCTGAATCACATGA	ENSGACT00000002893
	R: TGTGGCATTTAACAACATTTCCA	
*ar*	F: TGGTCTTCCTCAACATCCTG	ENSGACT00000042396
	R: ACCCTGGCAATCCTT	
*aqp10b*	F: GAGGTTTTCGAGGCTGGAG	ENSGACT00000005857
	R: TGACGTGGCATCTGTTCTTC	
*atp1a1*	F: AGGCGGAGTAGGAAAGTTGC	ENSGACT00000018949
	R: ATCTTTCCCTCTTCCCAGCC	
*cd2ap*	F: CCAAAAGAGGAGGCGCAGA	ENSGACT00000005627
	R: CCTCTTGGGCTTCTTGGGG	
*cldn15a*	F: ATGTGCACAATGGTTGCAGT	ENSGACG00000025514
	R: ACCTTATTCCGGGATGGAAG	
*kcnh4*	F: CACAGTGACCTCTCTGGTGC	ENSGACT00000011446
	R: AGACATGAGCAGGGTCAGGA	
*kirrel3 l*	F: TGACGGCGTTACACCTGATT	ENSGACT00000010235
	R: ACACAGGGTAGAGTCACGGA	
*slc12a1*	F: AGTTTGGCTGGATAAGGGGC	ENSGACT00000022179
	R: GCCTGACCGAAAATCCAGGA	
*spg*	F: CAGACATACACTTGCAGGACA	ENSGACT00000025232
	R: TCTCGACTTGGACCAACAGC	

### Data analysis

Venn diagrams were created for the kidney and the posterior and anterior intestines to show the unique and overlapping gene sets for each tissue using jvenn^47^. Biological functions, processes and pathways, and cellular components associated with transmembrane transport DEGs were identified using GO annotations, KEGG^48–50^and Reactome pathway analysis in STRING v 12.0 (http://www.string-db.org/)^51^. The input gene list includes transmembrane transport DEGs in the GO ontology of all DEGs and previously identified renal tubule transporters. Such an approach ensures the inclusion of previously uncharacterized renal tubule transporters. The resulting list is then uploaded as input on STRING. The analysis criteria were a combined score > 0.4, a maximum FDR ≤ 0.05, a minimum strength ≥ 0.01, and a minimum count in network 2. Bar graphs showing gene counts in the kidney and posterior intestine were individually created for solute transport DEGs differentially regulated between the two organs. Heatmaps showed significantly regulated solute transport genes in both the kidney and posterior intestine. Additional heatmaps were produced for aquaporins, tight junction proteins, and vesicle transport genes, irrespective of their regulation levels in both organs. Enriched GO terms were visualized using bubble plots created using an online data analysis and visualization platform^52^ (https://www.bioinformatics.com.cn/en). Terms and pathways were ranked based on their enrichment score and the top ten enriched terms are presented with the number of genes in each term represented by dots. The color gradient was used to indicate significance levels of the enrichment with the false discovery rate-based p-values. Heatmaps were generated for gene paralogs common to the kidney and posterior intestine.

### Statistical analysis

The GraphPad Prism 8.0.2 software (GraphPad Prism, USA) was used to determine the differences in the expression level of validation genes between castrated and 11 KA-treated tissues using one-way ANOVA followed by Dunnett’s post-test for multiple group comparison. Statistically significant differences were considered when p-values were < 0.05 (*p < 0.05, **p < 0.01 and ***p < 0.001). Principal component analysis (PCA) was generated for normalized gene counts using the PCA tool in Partek. To give equal weight to all features when computing the principal components, the ‘equally’ feature option was used in which all the features are standardized to a mean of 0 and a standard deviation of 1.

A number of significant components were validated using cross-validation rules. A tolerance ellipse based on Hotelling’s T2 was used to check for outliers (95%). For enrichment analysis, terms, and pathways with p-values < 0.05 were considered significant.

## Electronic supplementary material

Below is the link to the electronic supplementary material.

Supplementary Material 1

Supplementary Material 2

## Data Availability

The datasets generated and used in the current study are available in the NCBI repository, Sequence Read Archive (SRA) under the accession PRJNA1196704. Please contact the corresponding author for further information if necessary.
