# Supplementary material for: Functional transposition of renal functions to the posterior intestine during maturation in male three-spined stickleback
Source: Sci Rep. 2025 Jun 20;15:20164. doi: 10.1038/s41598-025-05513-z (PMC12181344; doi:10.1038/s41598-025-05513-z)
Supplement: Supplementary file 1 — Supplementary Material 1 [file 41598_2025_5513_MOESM1_ESM.docx]

Supplementary Figures

**Functional transposition of renal functions to the posterior intestine during nesting in male three-spined stickleback**

Yared H Bezabhe^1§^, Berkay Paylar^1§^, Asmerom Seyoum^1^, Bertil Borg^2^, Per-Erik Olsson^1^*

^1^The Life Science Center Biology, School of Science and Technology, Örebro University, 701 82 Örebro, Sweden

^2^Department of Zoology, Stockholm University, 106 91 Stockholm, Sweden

*Correspondence: Per-Erik Olsson, The Life Science Center, School of Science and Technology, Örebro University, 701 82 Örebro, Sweden.

Email: [per-erik.olsson@oru.se](mailto:per-erik.olsson@oru.se)

^§^These authors contributed equally


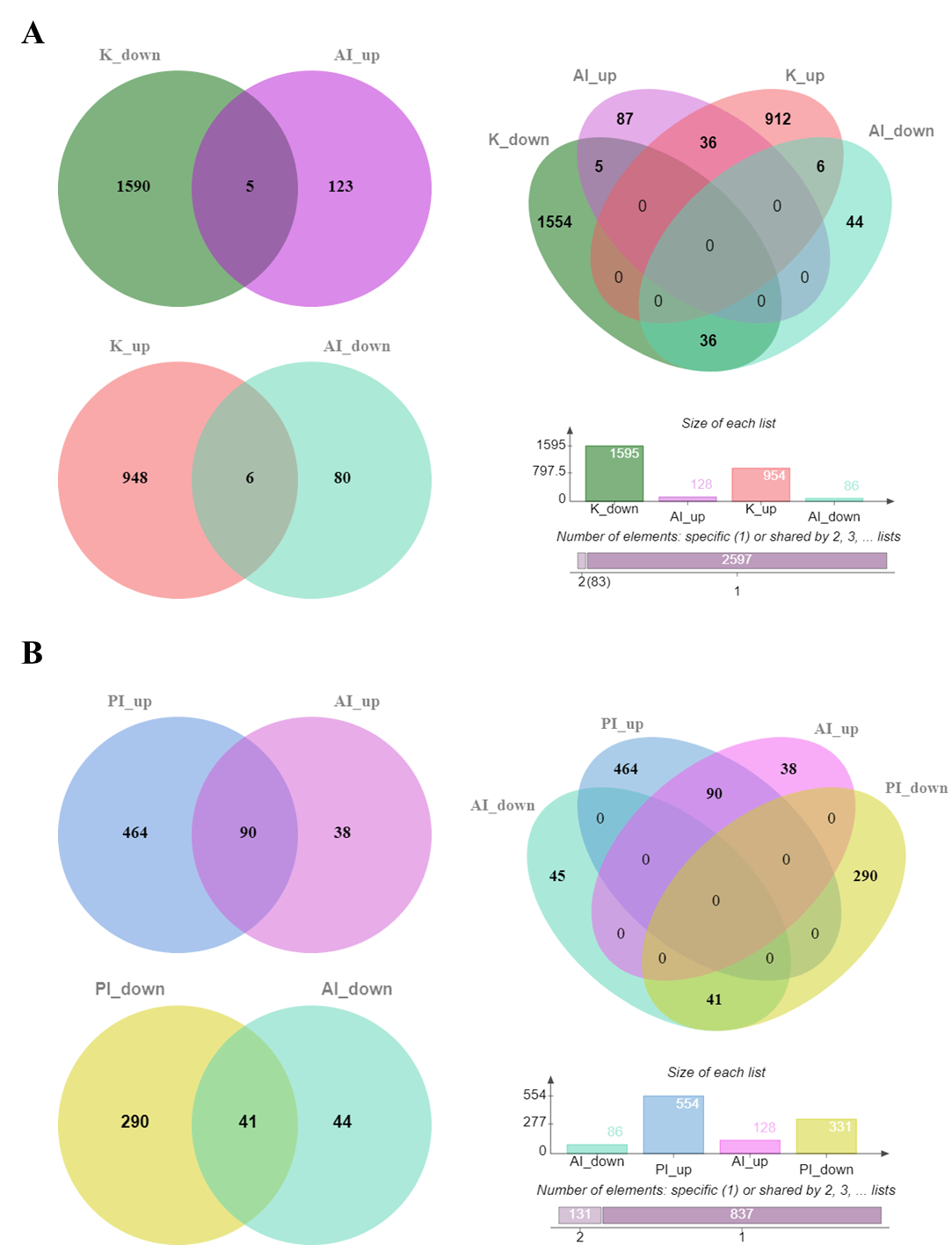


Fig S1. Venn diagram shows differentially expressed genes (DEGs) between (A) kidney and anterior intestine (B) Posterior and anterior intestine


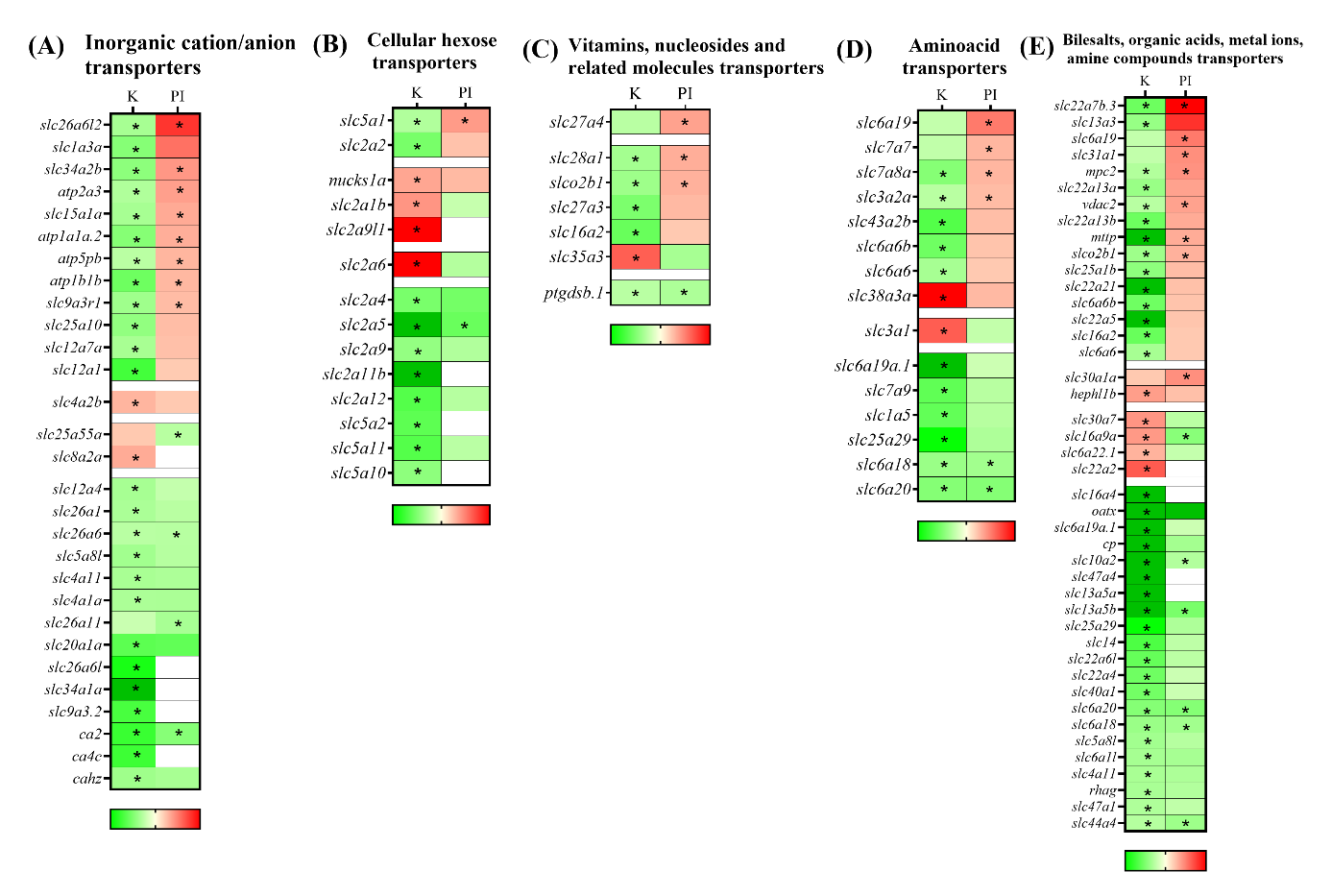


Fig S2. Heatmap showing the differential regulation of analogous and paralogs of solute transporters. (A) inorganic ion transporters (B) cellular hexose transporters (C) vitamins, nucleosides transporters (D) amino acid transporters (E) Bilesalts, organic acids, metal ions, amine compounds transporters. Genes showing significant fold changes between 11KA treated compared to castrated males in each organ were shown by red (upregulated) and green (downregulated) gradients (p<0.05, FDR≤0.15, FC ≤ -1.2 or ≥ 1.2). In each organ, non-expressed genes were shown by white shading. K-Kidney; PI-Posterior intestine.


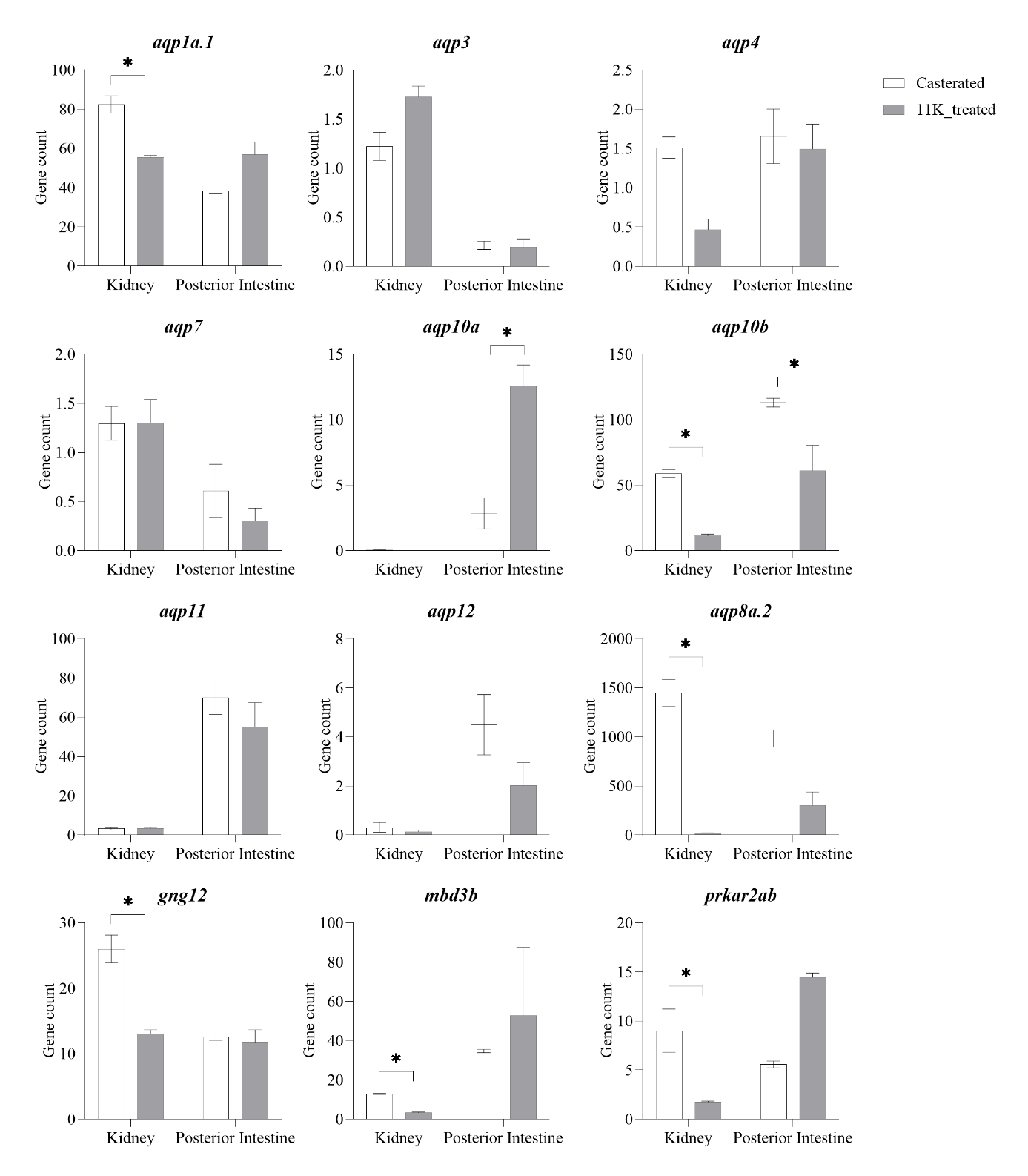


**Fig S3**. Gene counts of aquaporin paralog genes expressed in kidney. anterior and posterior intestine (*=p<0.05).

**
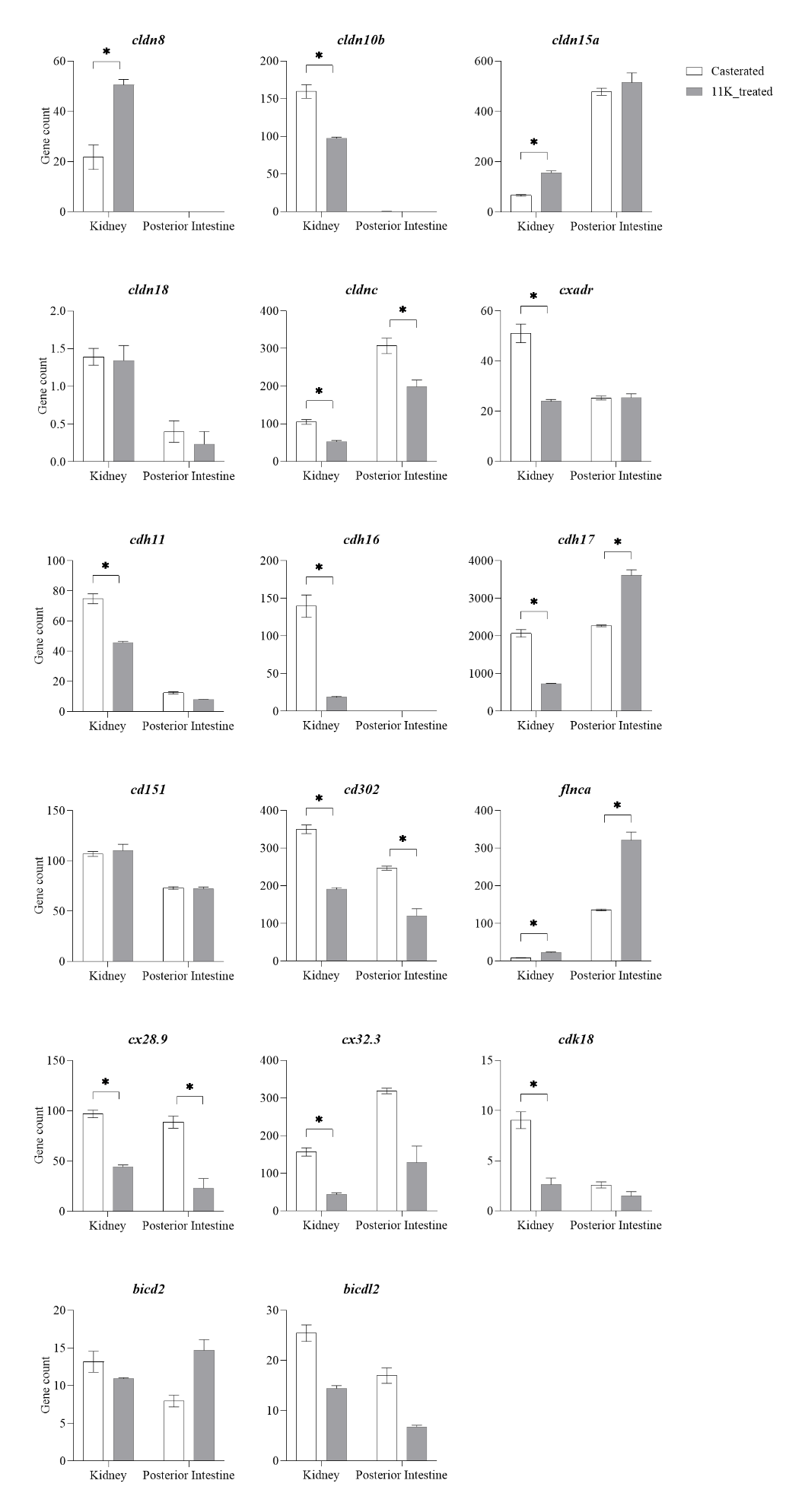
**

**Fig S4**. Gene counts of claudins, cadherins and connexins paralog genes expressed in kidney and posterior intestine (*=p<0.05).
